# Shining a Light on the Challenging Behaviors of Adolescents with Comorbid Diagnoses: Use of Pictorial Concurrent Operant Preference Assessment

**DOI:** 10.3390/children8080683

**Published:** 2021-08-08

**Authors:** Zhichun Zhou, David Burrell McAdam, Deborah Ann Napolitano, Kathryn Douthit

**Affiliations:** 1 Department of Counseling and Human Development, University of Rochester, Rochester, NY 14627, USA; david_mcadam@urmc.rochester.edu (D.B.M.); kdouthit@warner.rochester.edu (K.D.); 2 School of Education, Webster University, Webster Groves, MO 63119, USA; 3 Department of Pediatrics, University of Rochester School of Medicine and Dentistry, Rochester, NY 14642, USA; 4 Department of Applied Behavior Analysis, Daemen College, Amherst, NY 14226, USA; dnapolit@daemen.edu; 5 Golisano Institute for Developmental Disability Nursing, St. John Fisher College, Rochester, NY 14618, USA

**Keywords:** pictorial concurrent operant preference assessment, functional behavior assessments, behavioral functions, challenging behaviors, comorbid diagnoses, adolescents

## Abstract

Currently, there are no published studies that have used the concurrent operant preference assessment procedure to identify functions of challenging behaviors displayed by individuals with comorbid diagnoses. Four participants (aged 11–16 years) with comorbid diagnoses who displayed multiple challenging behaviors were referred to this study. We modified the standard concurrent operant preference assessment and used the new modified version, the *pictorial concurrent operant preference assessment*, to identify the functions of the challenging behaviors. Utilizing the triangulation mixed-methods design, we compared the indirect functional behavioral assessment (FBA) and the direct FBA with the pictorial concurrent operant preference assessment. The results obtained successfully demonstrated the concordance among these assessments in identifying the behavioral function for each participant. The results further showed that (1) the preferences served the same functional effects on both the challenging behaviors and the adaptive behaviors and (2) the pictorial concurrent operant preference assessment can be used independently to identify potential behavioral function and to specify the reinforcing potency of each behavioral function. The significance of the study results, limitations of this study, and directions for future research and clinical practice are discussed.

## 1. Introduction

Individuals with neurodevelopmental disorders (ND) [1], such as intellectual disabilities, autism spectrum disorder (ASD), and attention-deficit/hyperactivity disorder (ADHD), are prone to comorbid mood disorders. According to the *Diagnostic and Statistical Manual of Mental Disorders* [1], nearly 70% individuals with ASD may have a comorbid diagnosis of anxiety or depressive disorders. Additionally, children and adolescents with ADHD share symptoms of inattention with anxiety disorder and show significant changes in mood that could be a clinical indicator of bipolar disorder [1]. It is important to note that individuals with comorbid diagnoses of ND and mood disorders are more likely to exhibit challenging behaviors than their same-age typically developing peers [2,3]. Due to delays in expressive language, challenging behaviors often serve a communication function in individuals with ND [4]. According to the published literature, the most frequently observed topographies (i.e., physical forms) of challenging behaviors in community and educational settings are aggression, self-injurious behavior (SIB; e.g., hitting one’s own head, biting one’s own hand, pulling out one’s own hair), stereotypic behavior or repetitive behavior (e.g., body rocking, hand flapping), non-compliance, and disruptive behavior (e.g., property destruction).

Research has shown that challenging behaviors displayed by individuals with comorbid diagnoses of ND and mood disorders potentially result in a wide variety of negative consequences related to quality of life [5]. For example, challenging behaviors often interfere with opportunities to fully participate in educational programs and may limit opportunities for developing positive social relationships with same-age peers and community members. Additionally, the display of challenging behaviors may contribute to this population being either unemployed or under-employed after the completion of their education. Challenging behaviors are also reported to increase the stress level of direct-caregivers and/or family members [6,7]. Finally, challenging behaviors may expose direct-care providers and/or family members as well as the individuals themselves to potentially dangerous situations (e.g., serious episodes of challenging behaviors may require the support of community responders such as police officers; aggression may result in accidental injuries to direct-care providers or family members) [6,7,8]. 

In recent decades, a number of researchers have examined the prevalence of challenging behaviors in individuals with comorbid diagnoses of ND and mood disorders. However, the application of a variety of different operational definitions of challenging behaviors as well as research methodologies has resulted in significant differences in the outcome of published studies. For example, some researchers have focused on specific topographies of challenging behaviors, such as aggression [9], self-injurious behaviors (SIB) [10,11], or stereotypy [12], whereas other researchers have focused more broadly on challenging behaviors in general and have not limited the focus of their study to one or more specific topographies. In these research studies, challenging behaviors are used as a broad umbrella term for multiple combined topographies of challenging behaviors. For example, challenging behaviors might be defined as aggression, property destruction, non-compliance or repetitive behavior and participants might display any combination of these topographies of challenging behaviors [6]. Still other researchers have provided a very general definition of challenging behaviors (e.g., challenging behavior consists of any behaviors that are concerning to direct-care staff members) or have restricted their study population to a specific group, such as individuals with intellectual disabilities living in a residential facility [13], agencies or at home [14].

The variations in research methods have produced substantial differences in the prevalence of challenging behaviors. For example, Emerson and colleagues [15] sampled two areas of North Western England and found that 10–15% of individuals with multiple diagnoses exhibited challenging behavior, which represented a prevalence rate of 4.6 per 10, 000 of the total population sampled. Similarly, Lowe and colleagues [8] sampled 7 areas in South Wales and found that an average of 10% (range 5.5–16.8%) of individuals with learning disabilities displayed challenging behavior. Approximately 8% of the individuals who participated in this study displayed challenging behaviors at a level that caused serious concern on the part of direct-care staff and/or family members (e.g., an individual’s challenging behavior occurred at least once per day, required frequent physical intervention such as response blocking or restraint, and/or resulted in frequent injury to self or others). Due to the fact that these studies abovementioned were carried out in the UK, the data may not provide an accurate picture of the true overall prevalence of challenging behaviors of individuals with comorbid diagnoses in the US. Unfortunately, the overall prevalence of challenging behaviors in the US has not been as well examined as in Europe, most likely due to differences in how medical care services are provided and how medical information is collected [16].

Researchers in the US, particularly behavior-analytic researchers, have invested much time and effort to developing behavioral assessment procedures in hopes of obtaining a better understanding of the functions of challenging behaviors displayed by individuals with ND. As a result, there has been a significant advancement in behavioral assessments since the 1970s. Currently, functional behavior assessments (FBA) are considered to be gold-standard assessments [17]. As a core component of the scientific approach to the treatment of challenging behaviors, FBA has been empirically supported as a methodologically rigorous procedure when used to identify functions of challenging behaviors and develop function-based intervention plans for individuals displaying challenging behaviors. Unfortunately, the implementation of the FBA has posed barriers for care providers and professionals, such as school psychologists, because many of them have received minimal training in applied behavior analysis [17,18]. Additionally, the time commitment and the financial cost of functional-behavioral methods may also preclude caregivers, educators, or parents from requesting that a comprehensive FBA is conducted. Further, conducting functional behavior assessments may be particularly difficult for individuals with comorbid diagnoses of ND and mood disorders. For example, the analogue functional analysis itself may occasion high rates of non-compliance and aggression, especially under the conditions in which demands are placed on an individual or attention is withdrawn.

Hence, it would be clinically useful to examine alternative behavioral assessments for individuals with comorbid diagnoses that are effective at identifying functions of challenging behaviors, less time-consuming or costly to implement and less likely to occasion challenging behaviors. One such behavioral assessment is the concurrent operant preference assessment procedure that provides individuals with the opportunity to choose between actual activities, items, or situations [19]. The concurrent operant preference assessment is often a low-demand situation in which a high rate of attention is available, and there is a low likelihood for individuals to engage in challenging behaviors to avoid the assessment itself. Historically, behavior-analytic researchers have used the assessment procedure for two main reasons: (1) to identify potential reinforcers for challenging behaviors displayed by individuals with ND; (2) to improve the social validity of behavioral interventions to individuals with ND and their caregivers (e.g., stakeholders such as individuals with ND and/or their family members or direct-caregivers accept the importance of the results of behavioral assessments) [20,21]. Other than individuals with ND, the concurrent operant preference assessment procedure has much promise for individuals with comorbid diagnoses of ND and mood disorders [22]. 

To the best our knowledge, there are no studies to date that have used the concurrent operant preference assessment procedure to examine the functions of challenging behaviors displayed by individuals with comorbid diagnoses of ND and mood disorders. Additionally, it is unclear whether or not the preferences identified through the concurrent operant preference assessment could demonstrate the same functional effects on both the challenging behaviors and the adaptive behaviors assessed and observed in the FBA. It also is unclear to what extent the concurrent operant preference assessment would match the FBA results, and potentially predict the FBA results when used independently. 

**Figure A1:**
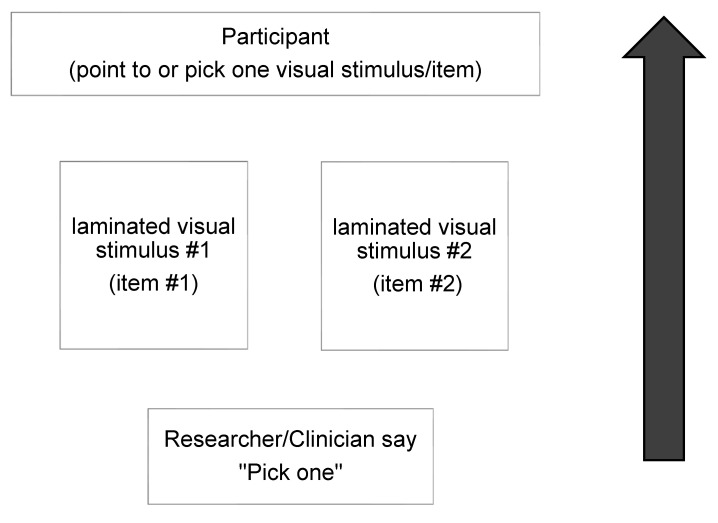
Explanatory diagram of the pictorial concurrent operant preference assessment procedure.

Therefore, the current study compares the FBA with the concurrent operant preference assessment to examine whether there is concordance between FBA and the concurrent operant preference assessment in identifying the functions of challenging behaviors of individuals with comorbid diagnoses of ND and mood disorders. In order to ensure the social validity of the concurrent operant procedure for this population, the procedure was modified in this study. One modification was the use of pictures combined with texts to present the actual social conditions/activities that may or may not routinely produce challenging behaviors. Another modification was the inclusion of a written stimulus/item “Your Choice”. The written stimulus “Your Choice” was added to (a) resemble the control condition outlined in the seminal study by Iwata et al. [23], in which participants have free access to social reinforcement (e.g., attention, tangibles, no demands) and to (b) assess whether the choice behavior itself could be a contingency of reinforcement. We call the modified version of the concurrent operant assessment procedure the pictorial concurrent operant preference assessment (see Figure A1). The assessment was conducted similarly to a traditional paired choice assessment [19]. The results obtained from the pictorial concurrent operant preference assessments were compared to the results obtained from the FBA.

## 2. Materials and Methods

### 2.1. Participants and Setting

This study was approved by the Research Subject Review Board at the University of Rochester (RSRB case number 00058241). Four participants with comorbid diagnoses of ND and mood disorders were referred to this study by their treatment team. It was reported that each participant frequently engaged in challenging behaviors in social environments (e.g., school and group activities). All participants were referred to by pseudonyms in this study. Danny, a 15-year-old boy, was referred for physical aggression and property destruction. John, a 16-year-old boy, was referred for physical aggression and trouble transitioning from school to residential home. Amy, a 15-year-old girl, was referred for elopement, physical aggression and non-compliance. Andrew, an 11-year-old boy, was referred for elopement, verbal and physical aggression, property destruction, and non-compliance. Table 1 listed the detailed demographic information and the behavioral topographies of each participant. The experimental sessions were conducted in three different group homes of a non-for-profit agency.

### 2.2. Procedure

There were two parts to the FBA procedure—the indirect FBA and the direct FBA. The indirect FBA included a session of the Functional Assessment Interview (FAI) and two sessions of the Questions About Behavioral Function in Mental Illness (QABF-MI). The direct FBA included three sessions of Antecedent Behavior Consequence (ABC) narrative recording. The pictorial concurrent operant preference assessment was conducted after the completion of the indirect and the direct FBA.

#### 2.2.1. The Indirect FBA—The Functional Assessment Interview

As a structured interview, the FAI [24] is one of the mostly commonly used indirect FBA methods in clinical settings. There are two main purposes of this interview: (a) identify and functionally define the target class of challenging behavior that is concerning to parents, guardians, and/or people who work closely with participants such as direct-care staff and teachers; and (b) gather information from parents, guardians, and/or direct-care staff about the distant setting events (e.g., sleep deprivation; medication) and the immediate environmental factors that may be attributed to development and maintenance of the target challenging behaviors [24]. The information obtained assists in the identification of the antecedent stimuli/events correlated with the target challenging behavior and the contingencies maintaining the target challenging behavior. That is, the behavioral function of the challenging behavior (e.g., social negative reinforcement in the form or escape or avoidance on instructional demands; social positive reinforcement in the form of access to high-preference tangible items). The FAI was conducted with the direct-care staff members who work closely with each of the four participants. The interview lasted 40 min on average. 

#### 2.2.2. The Indirect FBA—Questions about Behavioral Function in Mental Illness

The QABF-MI is a behavior checklist which asks 25 Likert scale questions about the behavioral function(s) of a single target behavior and is designed for caregivers, clinicians and educators who are most familiar with the target behavior to complete [25]. There are five questions per behavioral function, based on five potential behavioral functions, namely attention, escape, tangible, physical health, and non-social. Each question is rated on a five-point scale (0 = does not apply, 1 = never, 2 = rarely, 3 = sometimes, 4 = often). The results of the QABF-MI provide two sets of scores for each participant: (a) the severity score and (b) the endorsement score. The maximum severity score of the QABF-MI is 20. Each question receives a one-point endorsement score when the severity score is not zero. Thus, each potential behavioral function has a maximum endorsement score of five. The scale has been demonstrated to be a robust psychometric scale, which has adequate reliability (internal consistency *α* = *0*.84 − 0.92, and test–retest reliability *r* = *0*.86 − 0.99) and convergent validity (SMC = 0.69) [25,26]. 

One session of the QABF-MI was completed with the staff members who were most familiar with the participants, which lasted 10 min on average. In another session, each participant completed the QABF-MI for his or her own targeted behaviors, which lasted 25 min on average. It is worth noting that no studies to date have included the participants to complete the QABF-MI. We believe the inclusion of the participants themselves to the questionnaire would help us understand the target behaviors from the perspectives of the participants. To make sure the participants understood each question in the QABF-MI, the interviewer modified and simplified each question to match the specific participant’s scenario where challenging behaviors were observed. For example, the interviewer would ask, “The other day you hit a kid on bus, was it because you wanted his attention?”, or “You refused to get off the school bus, was it because you didn’t want to go back to the unit?”. 

#### 2.2.3. The Direct FBA—The ABC Narrative Recording

The ABC narrative recording was used to collect anecdotal information on each participant’s behavior repertoire and the environmental events/stimuli that may set occasion for a participant’s challenging behaviors. The narrative information was then analyzed qualitatively to examine whether specific antecedents and consequences are associated with a participant’s challenging and adaptive behaviors. Such qualitative analyses take into consideration of each participant’s behavior repertoire as well as the typographies of his or her behaviors, which compensate for the limitations experienced in the quantitative analyses used in the ABC continuous recording [27]. Although knowing the typographies of challenging behaviors may not statistically improve the accuracy of the identification of behavioral function, narrative records may provide an opportunity to systematically evaluate different environmental stimuli/events which may differentially affect the target challenging behaviors. Three sessions of the Antecedent Behavior Consequence (ABC) narrative recording were conducted with each participant within two to three weeks, and each session lasted approximately one hour for each participant.

#### 2.2.4. Pictorial Concurrent Operant Preference Assessment

The pictorial concurrent operant preference assessment was designed based on Fisher et al. [19]. This assessment was conducted to examine if it could be used in clinical and applied settings to identify potential functions of target challenging behaviors. Based on behavioral research standards, three sessions of the pictorial concurrent operant preference assessment were conducted across two to three weeks. Each session lasted 30 to 40 min. The items were identified by the participant him/herself and/or the direct-care staff who were most familiar with the participants. A total of 12 sample items were identified for Danny, John and Amy, and 13 sample items for Andrew. An item “Your Choice” was added as an independent control stimulus for each participant to assess if the choice behavior itself could be a contingency of reinforcement. Thus, the total number of sample items was 13 for Danny, John, and Amy, and 14 for Andrew. Each item was made into a color-printed laminated 2.5 × 2.5 inch card, with a brief textual description at its bottom. For example, one of the sample items identified by Danny was “play basketball”. The content of the laminated card was a colored visual image/picture of a boy playing basketball, with the text at the bottom of it that reads “play basketball”.

The items identified for each participant were randomized and sorted from the smallest to the largest in Excel (e.g., 1, 2, 3). Each item was paired with every other item and the pairs were written on index cards (e.g., 1 vs. 2, 1 vs. 3, 1 vs. 4). Since there were 13 sample items for Danny, John, and Amy, and 14 for Andrew, it rendered 78 trials for Danny, John, and Amy, and 91 trials for Andrew. During each assessment trial, the participant randomly chose an index card (e.g., 1 vs. 2), and the experimenter presented the two corresponding laminated pictures and verbally prompted the participant to “pick one.” A selection was scored when the participant physically touched or verbally described the content of one of the two items presented, after which both items were removed and the next trial started. The assessment continued until all possible pairs were presented once. Items chosen on 75% or more of the trials were designated as highly preferred; items chosen between 30% to 60% were designated as moderately preferred; items chosen on 15% or below were designated as least preferred [19,28]. The “Your Choice” card was chosen on 75% or more of the trials, and a further question—“what would be your choice?”—was asked. Because the referral indicated that all challenging behaviors were observed in social contexts, we categorized all items, except for the “Your Choice” card, into three categories of the social contingency: (a) social attention (e.g., talk to direct-care supporter and/or family members); (b) escape from demands (e.g., not being asked to do chores, no clean up); and (c) tangible items (e.g., listen to music, watch TV). 

### 2.3. Experimental Design

This study used a concurrent operant design to conduct the pictorial concurrent operant preference assessment. This design has been commonly used in published preference assessment research studies, e.g., [19,22]. A concurrent mixed-methods triangulation design [29] was used to compare the qualitative data obtained from the functional behavior interview (FBI) and the ABC narrative recording, and the quantitative data derived from the QABF-MI and the pictorial concurrent operant preference assessment. We chose this design because it fulfilled our research need of conducting the concurrent but separate collection and analysis of qualitative and quantitative data. 

### 2.4. Data Collection, Data Analysis, and Interobserver Agreement

Data were collected for challenging behaviors, adaptive behaviors and choice allocations. The measurement system included frequency, rate, duration and percentage chosen. Table 1 listed the specific topographies of the challenging behaviors and the adaptive behaviors. The summative content analysis [30] was conducted for the qualitative data obtained from the FBI and the ABC narrative recording. This specific qualitative analysis focuses on identifying and analyzing the effects of the environmental conditions on the target behaviors within the framework of the three-term contingency which comprises an antecedent condition (A), a challenging behavior (B) and a consequent condition (C). The environmental conditions identified through the interview and the narrative observations were further structured into the three potential socially medicated behavioral functions: (a) social attention; (b) escape from demands, and (c) tangible items. The results of the QABF-MI were analyzed using 10 as the cut-off severity score for each potential behavioral function in the behavior checklist (i.e., attention, escape, tangible, physical health, and non-social).

The data collected for the pictorial concurrent operant preference assessment were analyzed using visual inspection [31]. Each participant’s preferences derived from the assessment were displayed as percentage chosen in a rank order in bar charts and line graphs. The *X*-axes denote (a) the pre-identified sample items and (b) the assessment sessions, and the *Y*-axes denote (a) the percentage chosen allocated to the sample items in each assessment session, (b) the average percentage chosen allocated to the sample items of all three sessions, and (c) the average percentage chosen allocated to each of the three categories of the social contingency. The non-parametric statistical analysis, Kendall’s tau-b (τ_b_) correlation coefficient, was computed to access the strength and the direction of the associations among the three sessions of the pictorial concurrent operant preference assessment. 

In accordance with the concurrent mixed-methods triangulation design, we compared and analyzed the concordance of the assessments described above in identifying behavioral function for each participant.

Interobserver agreement was conducted to assess the direct functional assessment procedures, namely, the ABC narrative recording and the pictorial concurrent operant preference assessment. During each of these two assessment procedures, a secondary observer simultaneously but independently collected data for 33% of the sessions for purposes of inter-observer agreement. Agreements of the ABC narrative recording were defined as both observers having recorded the same rate of occurrence per hour. For example, observers counted the total number of adaptive and challenging behaviors within one hour, the rate of attention, demand and denied tangible occurred immediately before the behaviors (i.e., antecedents), and the rate of attention, escape from demand, and tangible access immediately after the behaviors (i.e., consequences). Agreements of the pictorial concurrent operant preference assessment were defined as both observers having recorded the same selection of item for each trial. Interobserver agreement for the ABC narrative recording was calculated by dividing the number of occurrence agreements by the number of agreements plus disagreements and multiplied by 100% [32]. Interobserver agreement for the pictorial concurrent operant preference assessment was calculated by dividing the number of agreements on the selection of item in each trial by the number of agreements plus disagreements and multiplied by 100% [19].

Following this, the interobserver agreements of the ABC narrative recording for Danny, John, Amy, and Andrew were 95%, 91%, 83%, and 92%, respectively. The interobserver agreements of the pictorial concurrent operant preference assessment were 97% for Danny and John, 100% for Amy and 96% for Andrew.

## 3. Results

### 3.1. Direct and Indirect Functional Behavioral Assessments

For Danny, the results of the FAI indicated that his property destruction and physical aggression were more likely to be occasioned and maintained by social negative reinforcement in the form of escape or avoidance of instructional demands and social positive reinforcement in the form of immediate attention from other people. The result of the QABF-MI obtained from the staff indicated Danny’s challenging behaviors served the function of escaping from demands and getting access to preferred tangible items, whereas the result from Danny himself showed that the behaviors served the function of receiving attention from others. Three sessions of the ABC narrative recording showed that Danny would display a high rate of adaptive behaviors (e.g., clean dishes, help to get mails, comply with demands, friendly manners) on the condition that he was given an immediate and high rate of positive reinforcement such as attention from peers and staff members and preferred items (e.g., music and basketball). The combined results of the FAI, the QABF-MI and the ABC narrative recording showed that both Danny’s adaptive and challenging behaviors served the purpose of producing the same reinforcement contingencies (i.e., social attention and tangible items), which suggests Danny’s behaviors (challenging and adaptive) belong to the same functional response class [33]. 

For John, the results of the FAI showed that he was more likely to display physical aggression and refusal when he was given a demand (e.g., asked to transition back to home or complete a task) or when his own demands were not met (e.g., he was not given access to the items he asked for). In comparison, the QABF-MI results provided by the staff member indicated John’s physical aggression and refusal were more likely to be occasioned and maintained by positive social reinforcement in the form of preferred tangible items. The QABF-MI session with John produced invalid outcomes due to attention drift (i.e., not focusing on the actual questions) resulting from John’s physical pain. Only adaptive behaviors were observed and recorded during the three sessions of the ABC narrative recording. The adaptive behaviors occurred mostly when John was not given any demands but provided with access to the tangible items he asked for (e.g., asking staff to switch TV channel to his preferred TV show) or to his preferred edible items (e.g., waffles and candies). This suggests that John’s adaptive behaviors were maintained by the conditions in which the least amount of response effort was required and access to preferred tangibles or edibles was freely available. The combined results of the FAI, the QABF-MI and the ABC narrative recording showed that both John’s adaptive and challenging behaviors served the purpose of producing the same reinforcement contingencies (i.e., no demand, access to the preferred tangible items that he preferred or asked for), which suggests that John’s adaptive behaviors and challenging behaviors belonged to the same functional response class. 

For Amy, the FAI results showed that all three categories of the social contingency (i.e., attention from staff members, escape from demands and tangibles) could set occasion for and maintain her physical aggression and elopement. The QABF-MI result obtained from the staff member showed Amy’s physical aggression and elopement were more likely to occur when she was not provided with attention and was denied access to preferred tangible items. The QABF-MI conducted with Amy showed that she was more likely to display challenging behaviors when she was asked to do something (e.g., told to go to bed, clean up, complete schoolwork) and denied access to the tangible items she liked (e.g., Netflix). Three sessions of the ABC narrative recording showed that Amy displayed elopement when she was not provided with one-on-one attention from staff members, and non-compliant behavior (e.g., walk away from schoolwork) when she was given a demand (e.g., complete schoolwork). These challenging behaviors were maintained by the same social contingency (i.e., immediate attention from staff members and teachers). 

For Andrew, the results of the FAI and the QABF-MI from the staff member demonstrated that Andrew’s challenging behaviors were more likely to be occasioned and maintained when he was given a demand or denied access to preferred tangible items. The QABF-MI session could not be completed with Andrew because he was not able to comprehend the scale questions. It was observed across the three sessions of the ABC narrative recording that in the presence of a high rate of social attention, Andrew’s challenging behaviors tend to occur as one hierarchical response class (e.g., starting from screaming cursing words and escalating to head banging, or starting from lying on floor and escalating to head banging). While in the situation where there was less or no attention, these behaviors would tend to occur independently as one single episode (e.g., screaming cursing words, or spitting at staff). The ABC narrative recording also showed that Andrew was more likely to engage in adaptive behaviors (chatting with staff, sitting quietly; compliant with daily routine) on the condition he was provided with attention from staff members and free access to preferred tangible items (e.g., video games; a preferred song). 

The combined results of the FAI, the QABF-MI and the ABC narrative recording showed that for both Amy and Andrew, their adaptive and challenging behaviors belonged to the same functional response class, which served to produce the same social contingency (i.e., no demand, immediate and high rate of attention, access to the preferred tangible items).

### 3.2. Pictorial Concurrent Operant Preference Assessment

**Figure 1:**
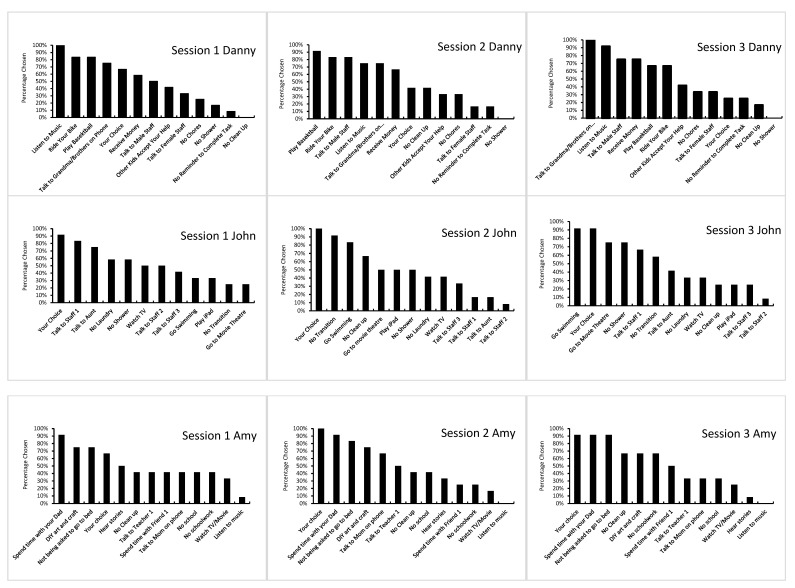

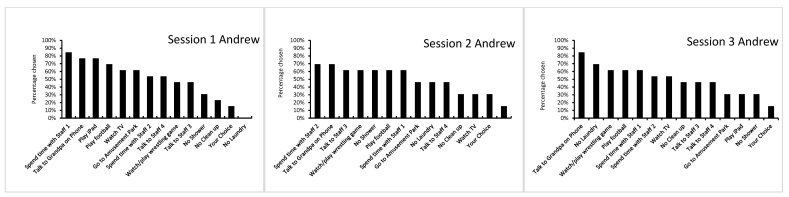
The percentage chosen allocated to each stimulus across the three sessions of the pictorial concurrent operant preference assessment for each participant.

Figure 1 illustrates the three sessions of the pictorial concurrent operant preference assessment conducted for each of the four participants. The results of the Kendall’s τ_b_ (see Table 2) indicated that for both Danny and Amy, there were statistically significant positive correlations among the three sessions of the assessment (*p* < 0.05 and/or *p* < 0.01). For John, the significant positive correlation was observed between session 2 and session 3 (r(13) = 0.44, *p* < 0.05). No significant correlation was observed among the three sessions of the assessment with Andrew.

**Figure 2:**
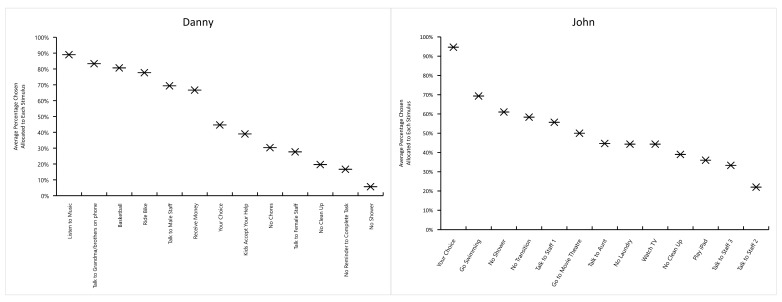

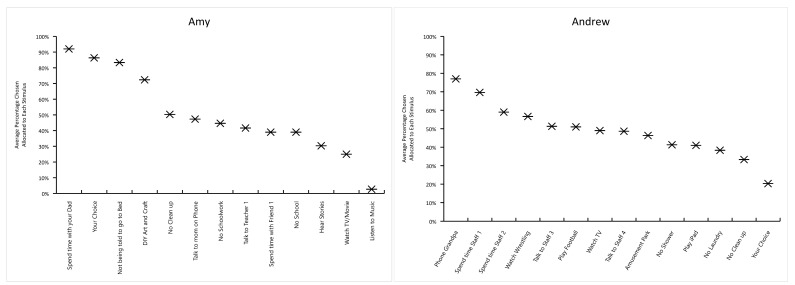
The average percentage chosen of the three sessions of the pictorial concurrent operant preference assessment for each participant.

Figure 2 illustrates the average percentage that each item was chosen after the three sessions of the pictorial concurrent operant preference assessment for each participant. Items chosen on 75% or more of the trials were designated as highly preferred; items chosen between 30% and 60% were designated as moderately preferred; items chosen on 15% or below were designated as least preferred. This resulted in Danny having four highly preferred items, with three items belonging to the social contingency of tangibles and one to attention. John only had one highly preferred item (i.e., the written item “Your Choice”). Amy had four highly preferred items, with each item representing a different social contingency (i.e., the written item “Your Choice”, social attention, escape from demands, and tangible items). The only highly preferred item chosen by Andrew was attention from a family member, followed closely by attention from a staff member. 

**Figure 3:**
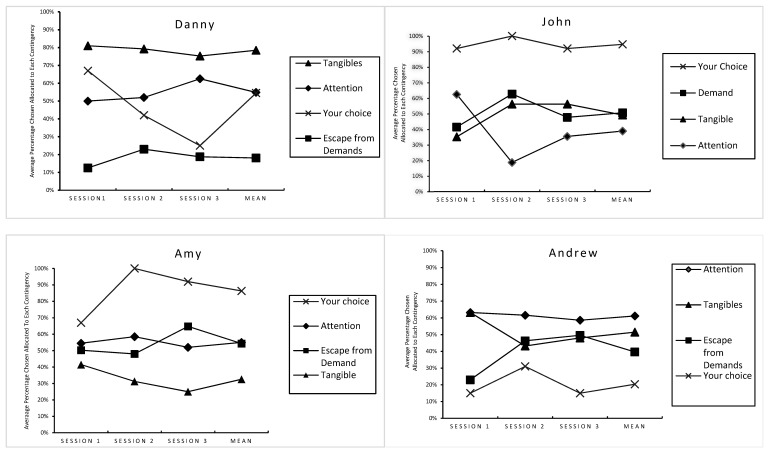
The average percentage chosen allocated to each category of the social contingency for each participant.

Figure 3 illustrates the average percentage chosen allocated to each social contingency (i.e., attention, escape from demands and tangible items) based on the categorization of the sample items identified for the pictorial concurrent operant preference assessment for each participant. Taking the mean of the results of the preferred social contingency across the three sessions of the pictorial concurrent operant preference assessment, Danny’s most preferred category of social contingency was the tangible items (M = 78.5%, SD = 2.9%), followed by the categories of social attention (M = 54.8%, SD = 6.7%) and escape from demands (M = 18.1%, SD = 5.3%). The item “Your Choice” was scored slightly lower than the category of social attention (M = 54.5%, SD = 21.1%). John’s most preferred category of the social contingency was escaping from demands (M = 50.7%, SD = 10.9%), followed by tangible items (M = 49.3%, SD = 12.1%) and social attention (M = 38.9%, SD = 22.1%). “Your Choice” remained high across all three sessions of his pictorial preference assessment, with the mean percentage chosen of 94.7% (SD = 4.6%). 

Amy’s most preferred category of social contingency was attention (M = 55%, SD = 3.3%), followed by the categories of escape from demands (M = 54.3%, SD = 9.1%) and tangible items (M = 32.6%, SD = 8.3%). “Your Choice” was scored higher than the three categories of social contingency (M = 86.3%, SD = 17.2). Social attention was also Andrew’s most preferred category of social contingency (M = 61.1%, SD = 2.3%), followed by escaping from demands (M = 39.6%; SD = 14.5%) and tangible items (M = 51.5%, SD = 10.4%). “Your Choice” was scored lower than the three categories of social contingency (M = 20.3%, SD = 9.2%).

### 3.3. Concordance: FBA and Pictorial Concurrent Operant Preference Assessment 

Table 3 demonstrates the concordance of the indirect FBA (i.e., the FAI and the QABF-MI), the direct FBA (i.e., the ABC narrative recording), and the pictorial concurrent operant preference assessment in identifying each participant’s behavioral function. The non-concordance (denoted as MISS in Table 3) only was observed within the FBA procedure. The rank order derived from the pictorial concurrent operant preference assessment further illustrated the specific and relative effects of preferences for each participant. That is, tangibles had relatively higher reinforcing value than attention on Danny’s behaviors. Escaping from demands had relatively higher reinforcing value than tangibles on John’s behaviors. Attention and escaping from demands had more relative reinforcing value than tangibles on Amy’s behaviors. Attention had relatively higher reinforcing value than tangibles and escape on Andrew’s behaviors.

## 4. Discussion 

The present study is the first published study that examines the theoretical understanding of the effects of preferences on challenging behaviors exhibited by adolescents with comorbid diagnoses through the use of the pictorial concurrent operant preference assessment. The results of this study provide a therapeutic starting point in developing behavioral treatments based on the effects of preferences. Additionally, the results of this study may promote the development of assessment protocols that are more clinically efficient and do not require the occasioning of challenging behaviors. 

The general positive finding is that there is concordance between the pictorial concurrent operant preference assessment and the FBA (i.e., the FAI, the QABF-MI, and the ABC narrative recording) in identifying behavioral functions. The concordance suggests that the pictorial concurrent operant preference assessment can be used as an alternative assessment in clinics to independently identify function(s) of challenging behaviors. The two ranking results of the pictorial concurrent operant preference assessment (see Figure 2 and Figure 3) provide specific information regarding the relative value of each social stimulus as well as the relative value of each social contingency. This suggests that when/if used in combination with the FBA, the pictorial concurrent operant preference assessment can be useful in interpreting the effects (i.e., reinforcing potency) of function(s) identified through the FBA procedure on behaviors. Additionally, it was demonstrated that challenging behaviors and adaptive behaviors can form one functional response class (i.e., they worked to produce the same social contingency). 

It should be noted that more meaningful information regarding each participant’s behavioral function(s) could be inferred from the results of this study. For Danny, the result of the direct FBA indicated that a high rate of adaptive behaviors was more likely to be observed on the condition that Danny was given immediate access to his preferred tangible items (i.e., music and basketball), and to a high rate of attention from staff members and peers. The finding was consistent with the phenomenon of matching law. Specifically, according to Herrnstein [34], the ratio of responses emitted between social reinforcers is controlled by the ratio of social reinforcers. However, in clinical and applied settings, we oftentimes observe the differences between the response ratio and the reinforcer ratio. Such differences may be the result of people’s constant preference towards certain parameters of reinforcers (e.g., rate or immediacy) [35]. Thus, in Danny’s case, he was more likely to display a high rate of adaptive behaviors when given immediate access to a high rate of social attention and preferred tangible items. It is reasonable to argue that Danny’s challenging behaviors would be occasioned if there was an unplanned overmatching between the adaptive social behaviors and the delivery of social contingencies [34]. That is, if there was a sudden and sustained change in the parameters (e.g., rate, immediacy, duration) of the social contingency (i.e., attention and preferred tangible items) that does not match the rate of Danny’s adaptive behaviors, Danny would display challenging behaviors(e.g., punching others, breaking property belongs to the residential home) to get an immediate and a high rate of the social contingency (i.e., attention and tangible items).

The results of John’s FBA showed that he maintained adaptive behaviors when he was in a highly reinforcing situation of his own choice. Thus, it is reasonable to infer that John would display challenging behaviors when he was not given what he requested. This inference was confirmed by the result of the pictorial concurrent operant preference assessment. Specifically, each time that John chose “Your Choice”, he preferred to “go outings” when further asked what “your choice” would be. This suggests that the item with the most reinforcing value was not certain specific tangible items, but an opportunity to choose to go anywhere that he requested. From the behavior analytic perspective, John’s challenging behaviors (i.e., refusal and aggression) were maintained by positive social reinforcement in a form of gaining access to high-preference community activities of his choice. In comparison, although Amy also picked “Your Choice” as the highly preferred item, she was uncertain of what her choice would be. The difference suggests that the two participants perceived “Your Choice” differently. In other words, their living and learning environment may have assigned different meanings to the two words, which in turn shaped their different perceptions to “Your Choice”. John perceived ‘Your Choice’ as an *actual opportunity* to choose the specific tangibles that he would prefer, whereas Amy perceived it as a *sense* of *freedom* to choose. 

In contrast, the other two participants, Danny and Andrew, did not allocate their choice to the “Your Choice” card on or more than 75% of the trials. From the developmental perspective, this may be attributed to the differences in the overall cognitive development, the age, and how the environment (e.g., caregivers, teachers, peers) shaped their perceptions towards the two words “Your Choice”. For example, both Danny and Andrew were diagnosed with intellectual disability, which might have impacted their capacity to comprehend the meaning underlying the two words. Additionally, the educational environment of Danny and Andrew might not have introduced the meaning of “Your Choice”, which may explain their low to moderate preference to the item “Your Choice”.

The inter-individual differences among the four participants also reflected in the consistency of their choice allocations (i.e., ordinal ranking of the stimuli presented) across the three sessions of the pictorial concurrent operant preference assessment. According to the results of Kendall’s tau-b (τ_b_) correlation coefficient, the choice allocations of Danny and Amy showed positive correlations among all three sessions of the assessment. In comparison, John’s choice allocations were positively correlated between two sessions. Additionally, no positive correlations were observed across the three sessions of the assessment with Andrew. The differences in the reinforcing potency and consistency of preferences might have their temperamental basis [36,37]. For example, Danny and Amy maintained high emotional and attentional reactivity to the same stimuli across all three sessions; whereas John maintained the similar level of emotional and attentional reactivity to the same stimuli across two sessions and Andrew did not maintain such a persistent reactivity to the same stimuli presented at all. It also is worth noting that none of the stimuli presented to Andrew evoked his high emotional and attentional reactivity (i.e., none of the items were chosen on or above 75% of all trials). 

Despite the inter-individual differences in the consistency of preference across the three sessions of the pictorial concurrent operant assessment, the results of the assessment were clinically significant in the sense that they enhanced our understanding that temperament may covary with the potency and consistency of preferences. Additionally, when comparing the average results of the three sessions of the assessment with FBA, we found the valence and intensity of participants’ emotional and attentional reactivity to the stimuli in one single session could not determine whether the stimuli would serve as functions of the participants’ challenging behaviors. 

The results of the ABC narrative recordings for Amy and Andrew demonstrated that regardless of the forms of antecedents (i.e., no attention or demands), the occurrence of their behaviors (challenging and adaptive) would increase when social attention was contingent on their behaviors. This finding suggests that social attention was a potent reinforcer for both of their challenging and adaptive behaviors. The specific finding corresponded with the results of their pictorial concurrent operant preference assessment, in which social attention was the most preferred category of the social contingency. Thus, it is reasonable to argue that social attention, as the most preferred social contingency, may also play a role as a conditional stimulus that could set the occasion for the behaviors of Amy and Andrew. Furthermore, as a conditional stimulus, social attention may have the same effect as a discriminative stimulus on their behaviors which were maintained by the same highly preferred social contingency, namely, social attention. This result served as the evidence supporting that the highly preferred social contingency could generate an equivalence relation (i.e., symmetric relation) between the conditional stimulus and the discriminative stimulus in terms of occasioning behavior [38].

**Figure 4:**
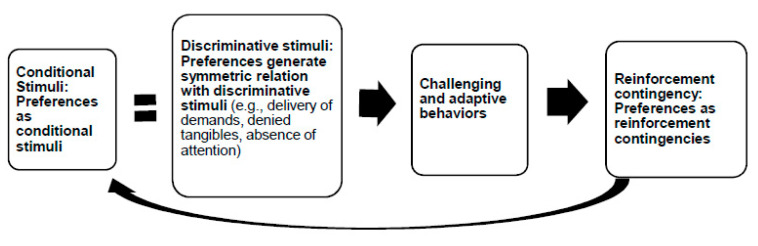
Multiple functions of high preferences in the behavioral four-term contingency: (1) high preferences serve as reinforcement contingencies; (2) high preferences serve as conditional stimuli as a result of the transference of their strong reinforcing value; (3) high preferences generate a symmetric relation between conditional stimuli and discriminative stimuli.

Figure 4 further explains the multiple functions of preferences in relation to behaviors described above. First, when the preference(s) with high percentage chosen score serves as a reinforcement contingency of behaviors (either challenging or adaptive), its reinforcing value could in turn have a strong effect as a conditional stimulus to occasion the behaviors. Such preference(s) could generate one of the equivalence relations (i.e., symmetric relation) between the conditional stimulus and the discriminative stimulus in the behavioral four-term contingency. The data for Amy and Andrew demonstrated that the delivery of demands (a discriminative stimulus) would have the same effect as attention (a conditional stimulus), in that both of them would occasion behaviors (e.g., Amy’s non-compliance behavior or Andrew’s aggressive behavior) that could produce the same reinforcement contingency, namely, highly preferred attention. Second, it was noted that when analyzing and combining the results of the ABC narrative recording and the pictorial concurrent operant preference assessment, the participants’ behaviors, either challenging or adaptive, were more likely to be strengthened (i.e., high frequency or duration) or to reoccur if the behaviors worked to produce the participants’ highly preferred items (e.g., attention, tangibles). In other words, if participants’ top-ranking (i.e., high scores on percentage chosen) preferences match exactly the social contingency produced by the participants’ behaviors, the behaviors are more likely to be maintained or to reoccur in the future. The specific finding regarding the effect of preference(s) on behavior mirrors Skinner’s argument that “[b]ehavior is often most vigorous and effective when an emotional predisposition works in the same direction as a contingency of reinforcement” [39] (p. 209). 

Several limitations of the current study should be discussed. First, we made an attempt to conduct a QABF-MI with each of the four participants in hopes of (a) collecting more information directly from the participant regarding the function(s) of their own behaviors and (b) avoiding the potential confounding variable such as emotional stress that the participants’ direct-care staff may have as a result of working with the participants. Unfortunately, the results from the two participants, John and Andrew, could not be used for data analysis due to John’s attention drift caused by his body pain and the undermatching between Andrew’s cognitive development level, age and the complexity of the questions. Second, there were inter-individual differences in their interpretations of the “Your choice” card and in the reinforcing potency and consistency of their preferences. Third, given the present study being single-subject design in nature, the sample size is small. In the behavioral analytic approach, the importance is placed on a detailed analysis of the effect of an independent variable at the level of the individual and the generality of findings is established through replications; thus, independent replication of the results reported in this study are necessary. Fourth, this study focuses solely on verifying the use of the pictorial concurrent operant preference assessment in identifying the function of the challenging behaviors. In the future, researchers should evaluate the clinical efficacy of function-based treatments based on the assessment results obtained from the pictorial concurrent operant preference assessment.

## 5. Future Directions

The results of the present study contribute to the existing literature on behavioral assessment in three ways. First, the overall results validated the value of using functional relation, rather than causal relation, as the conceptual system to explain the purpose of human behavior [40]. The combined results of the pictorial concurrent operant preference assessment and FBA revealed that when the behaviors (challenging or adaptive) worked to produce highly preferred items and categories of the social contingency (i.e., social attention, escape from demand, and tangible items), they were more likely to be strengthened or to reoccur. This is because the high reinforcing value of the preference also has a strong effect as a conditional stimulus. The preference as a conditional stimulus could either pair with a discriminative stimulus (as conditional discrimination) or replace a discriminative stimulus to elicit the behaviors (see Figure 4). In addition, for two of the participants, the discriminative stimuli of their challenging behaviors (e.g., delivery of demands or denied access to tangible items) showed a symmetric relation with the conditional stimulus (e.g., preferred social attention) in terms of eliciting different typographies of behaviors to produce the same highly preferred reinforcement contingency (i.e., preferred social attention). This particular result supports the argument that the reinforcement contingency generates an equivalence relation in the four-term behavioral contingency [38]. The effectiveness of applying the concept of functional relation to explain behavioral functions serves as convincing evidence against the notion that challenging behaviors exhibited by individuals with neurodevelopmental and mood disorders are simply products of their diagnoses [41]. Therefore, future researchers are strongly encouraged to replicate and extend the current paper to other age groups with comorbid diagnoses.

Second, the results of the pictorial concurrent operant preference assessment were analyzed in two ways: (a) preferences for specific sample stimuli within the three categories of the social contingency; and (b) preferences for each of the three categories of the social contingency (i.e., social attention, escape from demands, and tangible items). We did this for two reasons. First, knowing the ranking of the preference for a specific stimulus would decrease the false-positive result produced by the ranking of the preference for a certain category of the social contingency. For example, if social attention is a highly preferred category of the social contingency, it does not infer that all stimuli within this particular category would be more preferred than the stimuli in the less preferred category of the social contingency (e.g., escape from demand). It also does not infer that each item within this particular category has the same reinforcing potency. Second, the ranking of the preference for the categories of the social contingency would be more stable than the ranking of the preference for the stimuli within each category. Thus, analyzing preferences for the categories of the social contingency would increase the efficacy of the pictorial concurrent operant preference assessment, in that each category of the social contingency can include a variety of individual stimuli as long as each of these stimuli provides the same social effect on behaviors (i.e., gain social attention, escape from demand, and access to tangible items). 

Additionally, the concordance between FBA and the pictorial concurrent operant preference assessment suggests that the pictorial concurrent operant preference assessment can be used independently to assess the function of challenging behaviors and adaptive behaviors. The concordance also suggests that the pictorial concurrent operant preference assessment can be used for different purposes in clinics. Specifically, when used in combination with the FBA, the ordinal ranking of the preference for each of the three categories of the social contingency (i.e., social attention, escape from demand, and tangible items) can provide precise reinforcing value of the function(s) identified in the FBA procedure.

Thus, it is advised that future researchers and practitioners interested in behavioral assessments consider (a) ranking the results of the pictorial concurrent operant preference assessment by social stimuli and by categories of the social contingency, and (b) combining the pictorial concurrent operant preference assessment with the FBA procedure to precisely identify behavioral function(s) and the relative potency of each behavioral function. Further, due to the possibility that the potency and consistency of preferences across the three sessions of the pictorial concurrent operant preference assessment covary with temperament, it is advisable that future practitioners as well as researchers also integrate temperament assessment to the behavioral assessment package and conduct the pictorial concurrent operant preference assessment every three days to determine the precise reinforcing potency of each stimulus.

Third, “Your Choice” was a highly preferred item for two participants and was only a low to moderate preference for another two. One participant treated “Your Choice” as *an actual opportunity* to choose freely. Another participant treated “Your Choice” as *a sense of freedom* to choose, which was irreplaceable with any actual tangible choices. From the behavior-analytic perspective, the interpretation of “Your Choice” as an actual opportunity to choose may imply a form of control, as Skinner stated “…human behavior is [also] a form of control” [42] (p. 209]. The interpretation of “Your Choice” as a sense of freedom may be developed through social selection for the ontogenetic purpose [43,44]. This sense of freedom, as an ontogenetic behavior, becomes reinforcing over time because it is associated with the survival of individuals in the micro-environment that resembles an institution and a complex macro-environment that promotes individualism. 

Indeed, the finding on “Your Choice” may offer a clearest picture yet of behavioral functions. Specifically, the understanding of “Your Choice” is not homogeneous, which may be influenced by age, cognitive development, and how the environment assigns meaning to the two words. Further, the item “Your Choice” may serve not only as a measurement for the condition that simulates a high reinforcing environment (i.e., high attention, free access to tangible items and no delivery of demands), but also as a measurement for the construct of *private event* (i.e., unobservable internal status such as emotions) [39]. Therefore, future researchers are encouraged to design a measurement system to assess the level of potency that the item “Your Choice” has as a potential immediate reinforcer or as a conditional stimulus that serves to sustain and maintain human behavior. Future studies also could build on the finding of the present study regarding the different effects of the two words “Your Choice” on individuals. For example, researchers could develop a scale to scientifically measure whether or not “Your Choice”, to a broader population, serves more as a behavioral construct that directs human behavior or as an intangible but measurable philosophical concept that guides human behavior. 

## Figures and Tables

**Table 1:** Characteristics of participants.

Participant/Gender	Age	Speech	Diagnoses	Challenging Behaviors	Topographies	Adaptive Behaviors
Danny/M	15	Yes	Moderate Intellectual Disability; Intermittent Explosive Disorder	Property destruction; physical aggression	Breaking windows/doors; punching peers and staff	Greeting politely; getting mails; chatting with staff
John/M	16	Yes	ASD; Intermittent Explosive Disorder; Separation Anxiety Disorder; Oppositional Defiant Disorder; Mood Disorder-NOS; Bardet-Biedl Syndrome	Physical aggression; non-compliance	Refusing to get on the bus to residential homes; refusal of routine activities	Watching TV; saying “thank you”, “hi”
Amy/F	15	Yes	Oppositional Defiant Disorder; Depressive Disorder-NOS; ADHD	Elopement; physical aggression; non-compliance; property destruction	Walking off the building (school/unit) without permission; throwing objects at people; refusal of routine activities, whining; pulling fire alarm	Hearing stories; watching TV; chatting with peers and staff
Andrew/M	11	Yes	Intellectual Disability; Bipolar Disorder	Property destruction; physical/verbal aggression; physical harm towards self and others; non-compliance; elopement	Peeling wallpaper; throwing objects and spit at people; cursing; head banging; biting and kicking others; throwing himself on the floor, refusal of routine activities; running out of unit without permission	Watching TV; getting food for himself; dancing to the music with staff and peers in the unit

**Table 2:** Correlations of the three sessions of the pictorial concurrent operant assessment for each participant.

**Danny**			
	Session 1	Session 2	Session 3
Session 1	1		
Session 2	0.615 **	1	
Session 3	0.564 **	0.538 *	1
**John**			
	Session 1	Session 2	Session 3
Session 1	1		
Session 2	−0.282	1	
Session 3	0.179	0.436 *	1
**Amy**			
	Session 1	Session 2	Session 3
Session 1	1		
Session 2	0.667 **	1	
Session 3	0.564 **	0.641 **	1
**Andrew**			
	Session 1	Session 2	Session 3
Session 1	1		
Session 2	0.099	1	
Session 3	0.187	0.341	1

Kendall’s tau_b coefficients for correlations of the three sessions of each participant’s pictorial concurrent operant assessment. * Statistically significant at the 0.05 level (2 tailed). ** Statistically significant at the 0.01 level (2 tailed).

**Table 3:** Concordance among the pictorial concurrent operant preference assessment, the indirect FBA and the direct FBA in behavioral function.

Danny *	**Pictorial Concurrent Operant Preference Assessment**	**FAI**	**ABC**	**QABF-MI with Staff**	**QABF-MI with Danny**
Pictorial concurrent operant preference assessment	tangible > attention > escape				
FAI	attention and escape				
ABC	attention and tangible	attention			
QABF-MI with staff	escape	escape	tangible		
QABF-MI with Danny	attention	attention	attention	MISS	
John *	**Pictorial concurrent operant preference assessment**	**FAI**	**ABC**	**QABF-MI with staff**	
Pictorial concurrent operant preference assessment	escape > tangible > attention				
FAI	escape				
ABC	escape and tangible	escape			
QABF-MI with staff	tangible	MISS	tangible		
Amy *	**Pictorial concurrent operant preference assessment**	**FAI**	**ABC**	**QABF-MI with staff**	**QABF-MI with Amy**
Pictorial concurrent operant preference assessment	attention> escape > tangible				
FAI	attention, tangible, and escape				
ABC	attention and escape	attention and escape			
QABF-MI with staff	attention and tangible	attention and tangible	attention		
QABF-MI with Amy	escape and tangible	escape and tangible	escape	tangible	
Andrew *	**Pictorial concurrent operant preference assessment**	**FAI**	**ABC**	**QABF-MI with staff**	
Pictorial concurrent operant preference assessment	attention> tangible > escape				
FAI	tangible and escape				
ABC	attention, tangible, and escape	tangible and escape			
QABF-MI with staff	tangible	tangible	tangible		

* Matrix showing the concordant results among the pictorial concurrent operant preference assessment, the Functional Assessment Interview (FAI), the ABC narrative recording (ABC) and the Question about Behavioral Function-Mental Illness (QABF-MI) in behavioral function for each participant. The indirect FBA are the FAI and the QABF-MI. The direct FBA is the ABC.
